# Refactoring and performance analysis of the main CNN architectures: using false negative rate minimization to solve the clinical images melanoma detection problem

**DOI:** 10.1186/s12859-023-05516-5

**Published:** 2023-10-11

**Authors:** Luigi Di Biasi, Fabiola De Marco, Alessia Auriemma Citarella, Modesto Castrillón-Santana, Paola Barra, Genoveffa Tortora

**Affiliations:** 1 Department of Computer Science, University of Salerno, Fisciano, Italy; 2 Department of Computer Science, Universidad de Las Palmas de Gran Canaria, Las Palmas, Spain; 3 Department of Science and Technology, Parthenope University of Naples, Naples, Italy

**Keywords:** Melanoma, Skin cancer, Deep leaning, IoMT

## Abstract

**Background:**

Melanoma is one of the deadliest tumors in the world. Early detection is critical for first-line therapy in this tumor pathology and it remains challenging due to the need for histological analysis to ensure correctness in diagnosis. Therefore, multiple computer-aided diagnosis (CAD) systems working on melanoma images were proposed to mitigate the need of a biopsy. However, although the high global accuracy is declared in literature results, the CAD systems for the health fields must focus on the lowest false negative rate (FNR) possible to qualify as a diagnosis support system. The final goal must be to avoid classification type 2 errors to prevent life-threatening situations. Another goal could be to create an easy-to-use system for both physicians and patients.

**Results:**

To achieve the minimization of type 2 error, we performed a wide exploratory analysis of the principal convolutional neural network (CNN) architectures published for the multiple image classification problem; we adapted these networks to the melanoma clinical image binary classification problem (MCIBCP). We collected and analyzed performance data to identify the best CNN architecture, in terms of FNR, usable for solving the MCIBCP problem. Then, to provide a starting point for an easy-to-use CAD system, we used a clinical image dataset (MED-NODE) because clinical images are easier to access: they can be taken by a smartphone or other hand-size devices. Despite the lower resolution than dermoscopic images, the results in the literature would suggest that it would be possible to achieve high classification performance by using clinical images. In this work, we used MED-NODE, which consists of 170 clinical images (70 images of melanoma and 100 images of naevi). We optimized the following CNNs for the MCIBCP problem: Alexnet, DenseNet, GoogleNet Inception V3, GoogleNet, MobileNet, ShuffleNet, SqueezeNet, and VGG16.

**Conclusions:**

The results suggest that a CNN built on the VGG or AlexNet structure can ensure the lowest FNR (0.07) and (0.13), respectively. In both cases, discrete global performance is ensured: 73% (accuracy), 82% (sensitivity) and 59% (specificity) for VGG; 89% (accuracy), 87% (sensitivity) and 90% (specificity) for AlexNet.

## Background

Melanoma is one of the most common types of skin cancer worldwide. Following the American Cancer Society statistics ,^1^ 97,610 new melanomas will be diagnosed in 2023, with an incidence of about 58,120 in men and 39,490 in women. Furthermore, 7990 people are expected to die due to melanoma, including 5420 men and 2570 women. Melanoma represents only 1% of skin cancers, but it has a high mortality rate due to its capacity to spread fast and metastasis to numerous areas. The incidence and mortality rates of cutaneous melanoma vary significantly by country and gender; in fact, it is more common in white, older men, with a mean age at diagnosis of 65. The more effective treatment for prevention is the surgical removal of the primary tumour before tumour cells detach the lymph nodes, allowing the tumour to spread rapidly. Melanoma develops biologically from melanocytes, pigment-producing cells located in the epidermis, the most superficial layer of the skin [1]. Both genetic and environmental risk factors influence melanoma development. The possible environmental causes are: UV radiation revealed by sun exposure and subsequent sunburn, particularly before the age of 35 [2]; the presence of melanocytic or dysplastic naevi; a personal history of skin melanoma; a family history of skin melanoma; phenotypes such as blond hair, eye, and skin colour; the tendency to have freckles [3, 4]. The past decade has led to a detailed understanding of the genetic basis of melanoma [5]. In this situation, disease progression is related to the acquisition of gene mutations. Benign naevi frequently have only one activating mutation, most commonly BRAF (Val600Glu), which causes about 50% of melanomas. Additional events, such as TERT promoter mutations or CDKN2A loss, have been observed in borderline lesions [6, 7]. Although tests are available to check for gene mutations, these are not recommended because no clinical benefit has been established thus far. Early detection of melanoma is critical as it considerably reduces mortality in 90% of cases because it will enable therapeutic intervention at a less advanced stage when it is still localized to the site of tumour growth [8]. Furthermore, a study comparing risk-adapted specialized skin surveillance with regular skin screening shows melanomas are more likely to be discovered at an early stage [9]. Unfortunately, populations and screening procedures vary by country, and there are rarely clear criteria. For example, in Germany, regular skin cancer screenings are suggested for people over 35, whereas skin cancer screenings are generally not recommended in the United States. The absence of a standard protocol could lead to a failure in early detection. Patients may suffer negative effects from inaccurate or delayed diagnoses, particularly when most effective treatment outcomes depend on early detection. The absence of uniformity could contribute to variations in diagnostic procedures, which could result in inaccurate assessments and a misinterpretation of clinical images. It is crucial to work toward even improved sensitivity and accuracy in melanoma detection models as technology and machine learning algorithms advance in order to support medical diagnosis. Reducing the risk of false negatives is crucial in the context of melanoma detection because timely diagnosis plays a vital role in improving patient prognosis and treatment success rates.

### The stage of melanoma

**Fig. 1:**
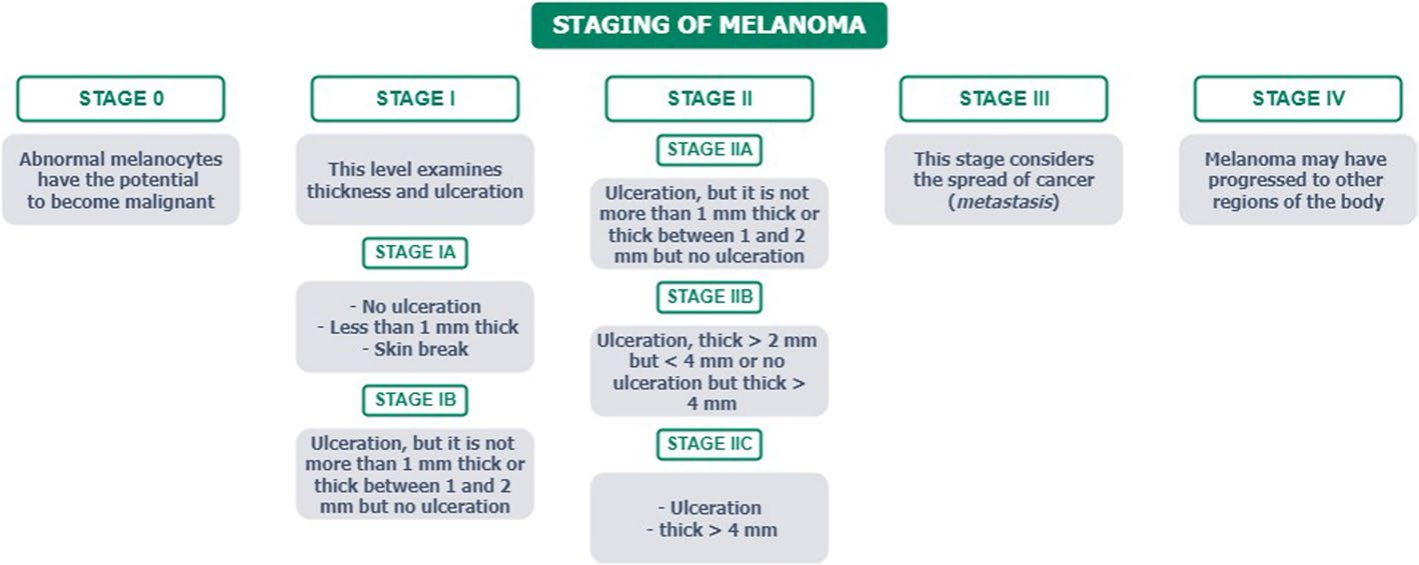
Melanoma staging diagram [10]

The stage of melanoma is determined by considering various visual, clinical, and biological features, including factors such as tumor thickness, ulceration, and the presence of metastasis in lymph nodes or other regions of the body, as reported in the *American Joint Committee on Cancer Staging Manual* [10], summarized in Fig. 1. Understanding tumor stages is crucial for evaluating treatment and prognosis. Nowadays, there are various invasive (less or few) tools able to identify the stages of melanoma, including lymph node mapping [11], Computed Tomography (CT) scan, Positron Emission Tomography (PET) scan [12], Magnetic Resonance Imaging (MRI), blood chemistry tests [13], and biopsy.

### The significance of computer-aided detection (CAD) in melanoma detection

With the development of artificial intelligence (AI) methods such as machine learning (ML) and deep learning (DL), it is now feasible to assist clinicians with a wide range of activities. In order to extract relevant data for digital health, these cutting-edge technologies are increasingly applied to biomedical challenges [14, 15], such as proteomics [16, 17], genetics and image and signal data classification [18, 19], and visualization [20]. Additionally, the Internet of Medical Things (IoMT), a subset of the Internet of Things (IoT) dedicated to the connectivity of all medical equipment, expands as more medical devices are connected [21]. As a result, new intelligence systems for health and well-being supported by mobile apps, robots, and remote servers such as in [22, 23] are possible. All of these scenarios pave the way for the application of these cutting-edge technologies for melanoma diagnosis.

In contemporary medical practice, a comprehensive people check-up typically involves a thorough examination of the skin of the entire, aided by techniques such as dermoscopy or other imaging methods. These examinations are carried out by experienced professionals. In cases where a potential risk naevus is identified by the expert, a biopsy is necessary to establish an accurate diagnosis of melanoma or non-melanoma. However, the standard approach faces two notable limitations. Firstly, the absence of an internationally recognized screening protocol for melanoma results in a lack of standardized datasets, hampering statistical and exploratory analyses. The manual nature of a full-body scan, often requiring the use of dermoscopy, can also prolong the process. Secondly, the screening process relies on human experts who apply their expertise and knowledge in evaluating the results.

In the event of any complications, requesting a biopsy for a benign naevus may become necessary, which could result in an invasive procedure for the patient. On the contrary, a biopsy may not be requested for melanoma. Considering the conditions mentioned above, in the first case, a type I error (false positive) occurs during verifying a statistical hypothesis when the true null hypothesis is incorrectly rejected. In contrast, in the latter case, we have a type II error (false negative), which is the failure to reject an incorrect null hypothesis. Following that, the *False Positive Rate* (FPR) and *False Negative Rate* (FNR) can be defined as the proportion of all negative results that lead to positive test outcomes and the proportion of positives that lead to negative test outcomes, respectively. Therefore, even though it is possible to collect and analyze the classification performance both for humans and CAD to understand who performs better, it is tough to improve human performance in a short time. At the same time, it is essential to improve CAD performance by increasing the training, validation, and test sets used. Recently, artificial intelligence techniques have been employed to classify melanoma and nevi and to assess the performance of these algorithms in comparison to the evaluations of dermatologists, reaching equally optimal results [24]. The Convolutional neural networks (CNNs) have been shown to provide the most accurate and precise results for constructing skin lesion classifiers [25, 26]: the significant improvement made by these results is that unnecessary biopsies are frequently avoided while needed biopsies are missed only a few times; this significantly reduces FNR and FPR.

**Fig. 2:**
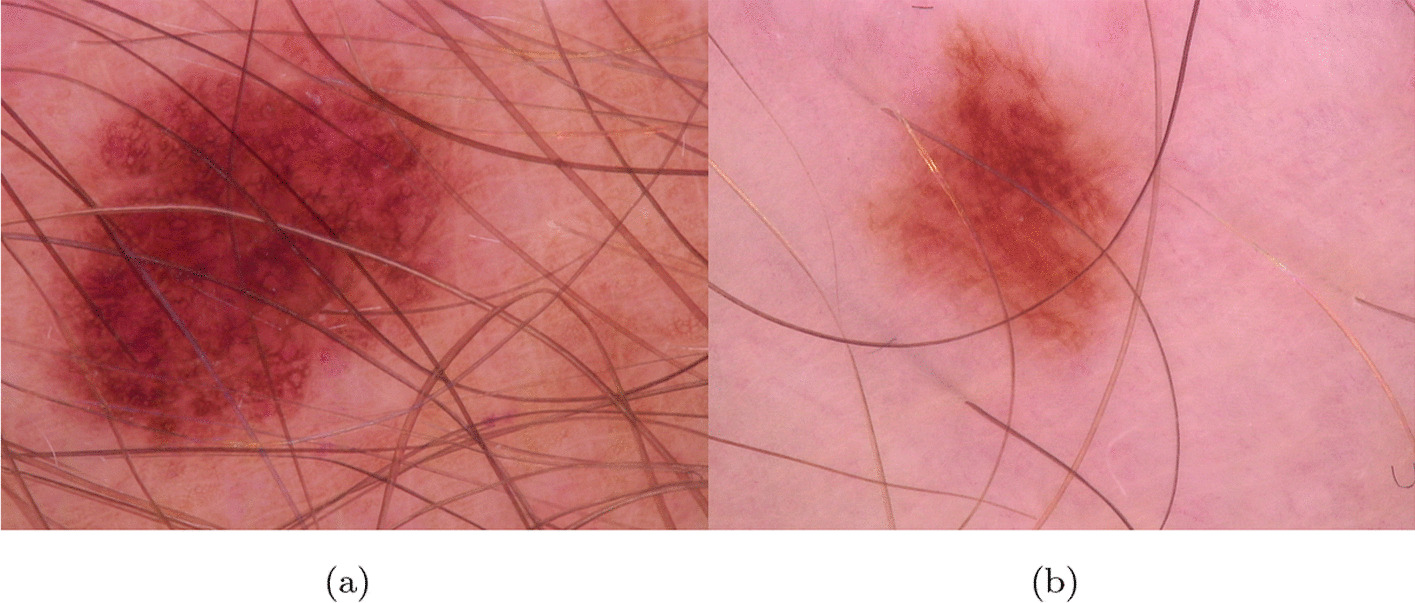
Dermoscopic images **a**, **b** from ISIC Archive

What is interesting to see is that even though clinical images are easy to capture and could provide similar performance as dermoscopic images [27], most of the works in the literature are based on the classification of dermoscopic images (see Fig. 2). This makes designing a CAD system hard due to the need to interoperate with a dermoscopic; also, this makes the CAD unusable in all the contexts where a dermoscopic could not be available. In the Internet of Things (IoT) and Internet of Medical Devices (IoMD) era, CAD system services should be provided to patients without their needing to visit the clinic physically or to have a dermoscopic at home. In particular, IoMD are all the interconnected network of medical devices and systems collecting, exchanging, analyzing health data for improved care, remote monitoring, and healthcare management.

Our goal is to allow patients to take an active role in their healthcare as digital health advances, enabling them to take images of their health issues, send them to online services, and quickly receive initial evaluations on whether they require further medical care. This digital strategy intends to improve patient accessibility, comfort, and rapid decision-making, ultimately expediting the healthcare process.

Consequently, we aimed to compare several CNNs to identify the best network, in terms of FNR, that could be eligible to be used for a CAD in the melanoma detection field using clinical images. We have chosen to consider FNR minimization because missing a needed biopsy on the skin is more dangerous (life-threating) than making a biopsy without melanoma. The neural networks studied, updated and trained are AlexNet [28], DenseNet [29], Google Inception V3 [30], GoogleNet [31], MobileNet [32], ShuffleNet [33], SqueezeNet [34] and VGG [35]. This paper is organized as follows: in the *Related works* section, we look at the present level of research on the health monitoring system for melanoma detection. Next, the applicable methodology is described in the *Methods* section. The dataset and detection approaches are then specified. Finally, the results acquired through a comparison with the literature are addressed in detail in the *Results and Discussion* section. The *Conclusion* section examines the challenges and future research directions for advancing the IoMT in melanoma surveillance.

## Related works

The vast majority of the works in the literature are based on the classification of dermoscopic images (see Fig. 2). In this section, we reported the most important works based on the use of clinical images of melanoma.

Nasr-Esfahani et al. [36] proposed using a deep learning system on a computer server equipped with a graphics processing unit (GPU) to detect melanoma lesions using clinical images. Clinical input images, which may involve illumination and noise effects, are pre-processed and then submitted to a pre-trained CNN that distinguishes between melanoma and benign cases in the proposed system. The collection consists of 170 non-dermoscopic images (70 melanoma, 100 naevi) from the University Medical Center Groningen’s Department of Dermatology’s digital image library (UMCG). The proposed system has reached 81% of accuracy.

The authors in [37] used a GoogLeNet DCNN model architecture trained on a dataset of clinical images of malignant melanoma (MM), squamous cell carcinoma (SCC), bowen disease, actinic keratosis, basal cell carcinoma (BCC), naevus cell naevus (NCN), blue naevus, congenital melanocytic naevus, spitz naevus, sebaceous naevus, poroma, seborrhoeic keratosis, naevus spilus and lentigo simplex. In particular, there are 540 malignant melanoma images, reaching an accuracy of 72.6%.

The study presented in [38] used a dataset of more than 12,000 skin images of malignant and benign tumors, from which they extracted 5846 clinical images of pigmented skin lesions from 3551 patients. The dataset contains 1611 malignant melanoma images. This study used a Faster Region-based CNN (FRCNN) model because it consistently demonstrated good classification accuracy, robustness, and speed. The authors evaluate the classification of FRCNN into six classes: malignant melanoma and basal cell carcinoma (malignant classes), naevus, seborrheic keratosis, senile lentigo, and hematoma/hemangioma (benign classes). They achieve an accuracy of 86.2%. The accuracy, sensitivity and specificity for two-class classification (benign or malignant) were 91.5 %, 83.3 % and 94.5%, respectively.

In [39], the authors used a dataset of 33,980 manually curated images, among them 296 are melanomas. Dermoscopy and nondermoscopy images were included for all pigmented-lesion classes in order to analyze both the types of images. Based on deep convolutional neural networks, their method achieved over 90.3% sensitivity and 89.9% specificity in distinguishing suspicious pigmented lesions from non-suspicious lesions, skin, and complex backgrounds. They also introduced a novel method to assess lesion saliency based on DCNN features, validated against dermatologists and reaching 82.96% of accuracy.

Ba et al. [40] proposed a multi-class CNN trained and validated using a dataset of 25,773 clinical images approved by the Chinese PLA General Hospital & Medical School’s Institutional Review Board. It covers ten types of skin cancer: basal cell carcinoma (BCC), squamous cell carcinoma (SCC), including keratoacanthoma, melanoma (MM), Bowen disease (BD), actinic keratosis (AK), melanocytic naevus (MN), seborrhoeic keratosis (SK), haemangioma, including pyogenic granuloma, cherry haemangioma, sinusoidal haemangioma and angiokeratoma, dermatofibroma (DF) and wart. CNN used in [40] achieved an overall accuracy of 78.45%, and CNN-assisted dermatologists achieved greater accuracy (76.60% versus 62.78%) than non-assisted dermatologists in interpreting clinical images.

In our previous work [41], we evaluated three neural architectures on the MED-NODE dataset: AlexNet, GoogleNet and Google InceptionV3. In this previous work, we addressed the issue of Transfer Learning (TL) and the development of a more adaptable system design that can accommodate changes in training datasets. Our findings suggest that AlexNet is the most robust network in terms of TL, without data augmentation, with mean accuracies of 78% and 89% with and without Otsu segmentation, respectively [42].

CNNs assistance improved the dermatologist’s accuracy in interpreting skin cancers and may increase the acceptance of this new procedure further. A recent systematic review explores 19 studies comparing classifications between CNN-based classifiers for melanoma, which show superior or equivalent performance to clinicians, regardless of the type of input data [43].

## Methods

### Dataset preparation

In this work, we used the dataset presented in developing the MED-NODE computer-assisted melanoma diagnosis system, called in this document MED-NODE [44] as the primary image source. The original dataset was made up of 170 clinical photos from the digital image archive of the Department of Dermatology at the University Medical Center Groningen (UMCG). There were 70 images of melanoma and 100 images of naevi.

To extend the training dataset size, we applied multiple combinations of image operators to the original dataset: data augmentation (DA), and image optimization. DA can aid in the extension of small datasets and the improvement of prediction performance.

In particular, we made three new training sets from the original MED-NODE dataset by applying different image operations and (DA) operators to the same images in different ways.

The operators we used to perform data augmentation to build the new dataset were: random rotation, random scaling, and random translation on X and Y. With these operators, we built a new dataset named “NSA” containing the MED-NODE original images and the new images generated by the DA operations applied to the MED-NODE original images.

Using NSA, we could compare the Neural Network (NN) performance to understand how data augmentation impacts NN prediction performance in this specific case of Melanoma Clinical Image Binary Classification Problem (MCIBCP). The results of the comparison are available in the following sections. We were also interested in evaluating the impact of the image quality improvement techniques on NN classification performance; in particular, we used the pre-processing quality step (IIQ) and a simple segmentation process (OTSU). More details regarding these two techniques are available in the next subsection.

From the original MED-NODE dataset, we built the following new datasets:

- “INA”,which contains MED-NODE original images improved by combining IIQ and the OTSU method (IIQpOTSU);
- “IA”, which contains NSA images improved by combining IIQ and the Otsu method (IIQpOTSU).

For clarity, the acronyms used to identify each dataset can be interpreted as:

- “INA”, containing MED-NODE original images by using quality improved and data augmentation techniques;
- “NIA”, containing MED-NODE original images not quality improved but using data augmentation techniques;
- “IA”, containing the NSA images by using quality improved and data augmentation techniques;

For coherence, we renamed the original MED-NODE dataset into NINA (Not improved, Not Data Augmented) in the following sections.

**Fig. 3:**
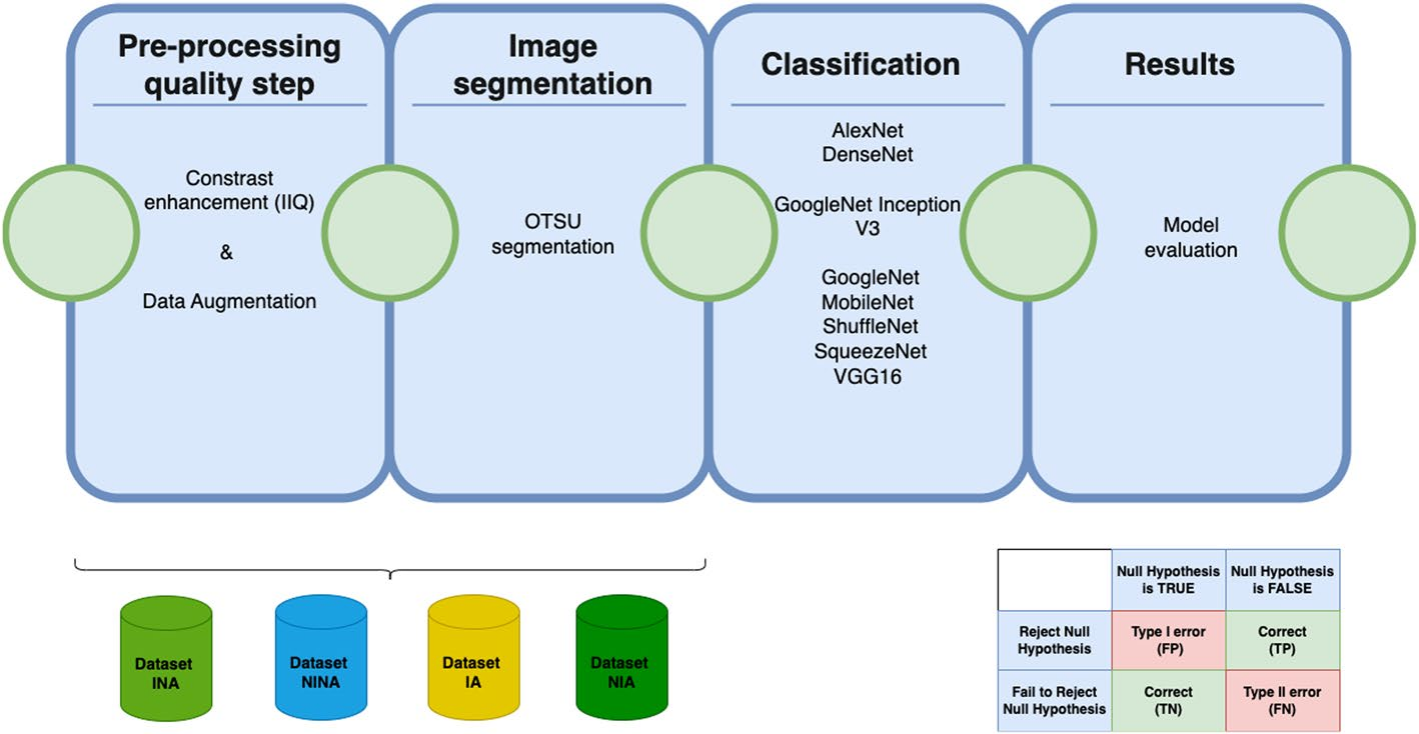
Workflow of the work

In Fig. 3, we graphically represented the workflow of our work.

### Image improvement method

Clinical images often suffer from poor contrast. To improve the quality of the MED-NODE, we used a MATLAB routine, histogram optimization, which enhanced the contrast of colored images. Image enhancement means improving an image’s perceptibility so that the final product is superior to the original: Image contrast enhancement before further pre-processing can improve analysis results [45].

**Fig. 4:**
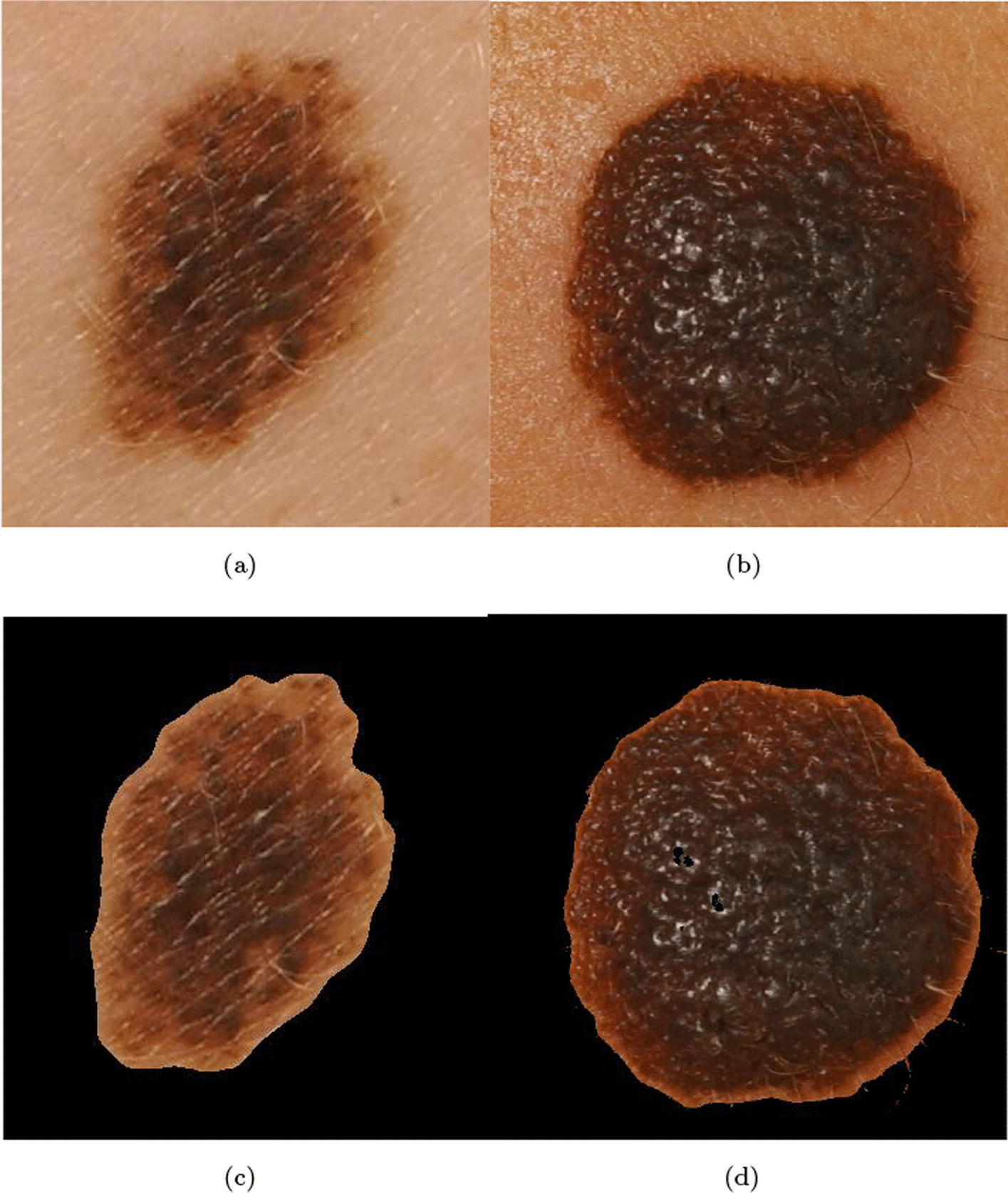
Naevi images before (**a**, **b**), and after (**c**, **d**) IIQ application

**Fig. 5:**
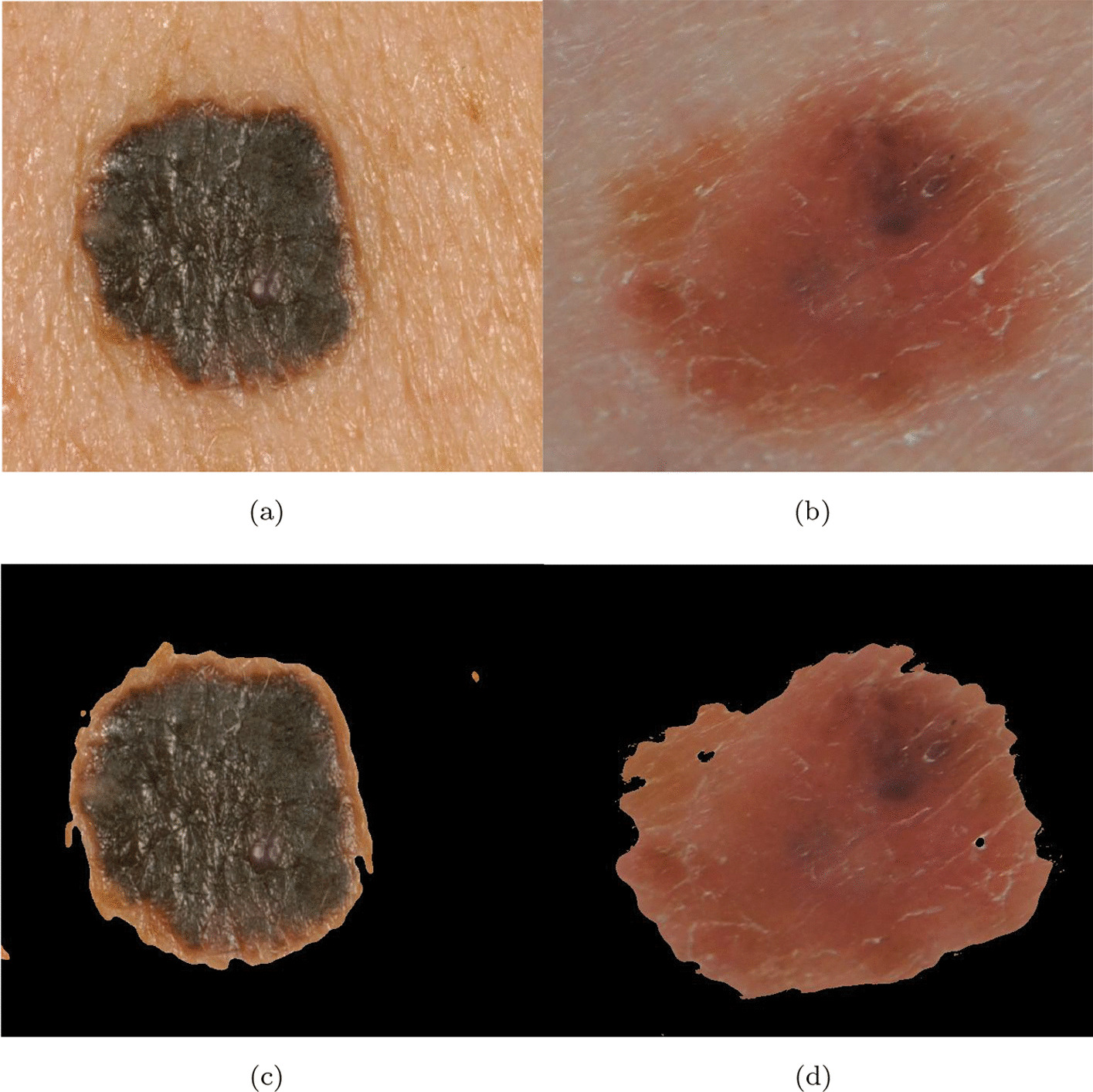
Melanoma images before (**a**, **b**), and after (**c**, **d**) IIQ application

In Figs. 4 and 5, a sample image before (a) and after the *IIQ application* (b) is shown for naevi and melanoma images, respectively.

### Image segmentation method

To investigate how segmentation might impact training performance, we used the OTSU method for the segmentation process. In particular, we used OTSU to make two of the three datasets described in the next section. OTSU performs automatic image thresholding, separating the pixels into background and foreground [46].

### CNN refactoring and evaluation

In order to identify the CNN that ensures the best FNR regarding the MCIBCP, we refactored and evaluated the performance of the following networks: Alexnet, DenseNet, GoogleNet Inception V3, GoogleNet, MobileNet, ShuffleNet, SqueezeNet and VGG16.

The original versions of CNNs come with pre-trained weights to solve a multi-class classification problem. In particular, these networks were trained on ImageNet [?] and can discriminate between 1000 classes of objects. As a first step, we discarded all the pre-trained weights from these networks. These preliminary steps removed all possibilities of transfer learning from the ImageNet upon which all these networks were pre-trained. Also, all the final layers (softmax, Fully connected) of all the CNNs were changed to allow these networks to discriminate between two classes instead of one thousand classes.

In [42], we reported results that strongly suggested that the training and validation steps could suffer from intra-class dissimilarities and extra-class similarities. In particular, we rely on the hypothesis that the CNN performance can vary, even if the training, validation, and test sets vary minimally. This fact can be observed in [47] when the ISIC 2018 winning algorithms performance dropped to a coin flip performance by only adding a new object class.

In order to avoid biased results, we followed a similar training protocol used in [42] to consider the mean performance instead of absolute performance to make our analysis more robust. The experimental environment used was MatLab 2021b .^2^

We performed 100 training steps for each network and dataset: 3200 (training, validation, and test) steps were performed to collect the experimental data. Each training step was performed by using MaxEpochs=30, MiniBatchSize=32 and InitialLearnRate=1e^−4^. For each training step, the training, validation, and test sets were allowed to change slightly while the previous network weights were discarded. No transfer learning was allowed during the training steps. The dataset was divided using the following ratios for each iteration: 0.5 for the training set (85), 0.3 (51) for the validation set, and 0.2 (34) for the test set. The randomized option of splitEachLabel method was enabled. The training set was split equally between melanoma and non-melanoma photos, chosen randomly from the starting image collection for each cycle. In addition, each image was resized to fit the network’s input constraints. For example, for Google Inception V3, the input images were resized to 299x299, while for AlexNet, the images were resized to 227x227 pixels. The training and validation sessions were executed using the trainNetwork function, while the classify function executed test sessions.

### Performance measurement

In order to evaluate the CNN performance in the MCIBCP context, we used the classical metrics such as Accuracy (ACC), Sensitivity (TPR, indicated as SN), Specificity (TNR, indicated as SP), Precision (PPV), False Discovery Rate (FDR), FNR and FPR.

We described them by the equations below (Eqs. 1–7):

1 $$Accuracy = \frac{{TP + TN}}{{TN + FP + FN + TP}}$$

2 $$Sensitivity\;\left( {TPR} \right) = \frac{{TP}}{{TP + FN}}$$

3 $$Specificity\left( {TNR} \right) = \frac{{TN}}{{TN + FP}}$$

4 $$Precision\left( {PPV} \right) = \frac{{TP}}{{TP + FP}}$$

5 $$FDR = \frac{{FP}}{{FP + TP}}$$

6 $$FPR = \frac{{FP}}{{FP + TN}}$$

7 $$FNR = \frac{{FN}}{{FN + TP}}$$

where TP and TN are the numbers of correctly predicted true positives and true negatives, whereas FP and FN are the numbers of incorrect predicted false positives and false negatives, respectively. The degree to which the measured value of a quantity corresponds to its true value is known as *accuracy*. The *sensitivity* of a test refers to its ability to detect true positives. Finally, the ability of a test to detect true negatives is measured by its *specificity*. It is important to note that in the MCIBCP context, we consider the specificity and the FNR, described below, as the primary and most essential metrics due to our goal to identify which technology can minimize the type 2 error.

*Precision* is a statistical measure that shows the percentages of true positive values in a test. The *FalseDiscoveryRate* measures the frequency of type I errors in null hypothesis testing.

## Results and discussion

In this section, we present the results collected in each experiment. As a first step, we reported the average, maximum, minimum, and standard deviation values for the ACC to identify which CNN performs globally better. However, we tried to present the results in a form that emphasizes the importance of having the lowest FNR possible in early melanoma detection.

Table 1 reports the ACCs of all the CNNs using the “IA” and “INA” datasets. We displayed the ACCs obtained with and without data augmentation, while imaging optimization techniques are consistently employed. The best mean accuracy results for the AlexNet and SqueezeNet networks are highlighted in bolditalic, settling at 78%. These findings highlight how these two CNNs might be the most resistant to inter-class/extra-class issues. It is interesting to see that Google InceptionV3, GoogleNet, and VGG reach an average accuracy greater than 70% when the data augmentation is not used on the INA dataset. Overall, all tested CNNs perform better on the INA dataset, suggesting that data augmentation using scaling, rotation, and translation may reduce classification performance. Again, AlexNet obtained the best global performance. Artifacts are unintended changes or distortions introduced into data. These results suggest that the most important effort could be dedicated to mitigate artifacts to ensure the reliability and validity of the analysis.

**Table 1:** The global performance of the CNNs on the INA and IA datasets are reported. In addition, image improvement techniques are active

IIQ active					
Net	Data augmentation	ACC (min)	ACC (max)	ACC (mean)	ACC (SD)
*AlexNet*	None	0.65	0.94	***0.78***	0.06
	Yes	0.44	0.91	0.68	0.08
*DenseNet*	None	0.56	0.79	0.69	0.05
	Yes	0.41	0.85	0.66	0.12
*Google InceptionV3*	None	0.56	0.94	0.76	0.07
	Yes	0.32	0.74	0.53	0.09
*GoogleNet*	None	0.60	0.91	0.75	0.07
	Yes	0.32	0.74	0.55	0.09
*MobileNet*	None	0.47	0.79	0.58	0.04
	Yes	0.35	0.74	0.49	0.09
*ShuffleNet*	None	0.53	0.82	0.66	0.06
	Yes	0.15	0.74	0.50	0.11
*SqueezeNet*	None	0.65	0.91	***0.78***	0.05
	Yes	0.35	0.79	0.58	0.09
*VGG*	None	0.59	0.83	0.74	0.05
	Yes	0.53	0.79	0.70	0.05

Table 2 reports the ACCs of all the CNNs using the “NINA” and “NIA” datasets. In this case, no IIQ techniques are active.

AlexNet reaches a mean accuracy of 89% when no data augmentation is used. GoogleNet settled on 80%. Again, the results suggest that the best outcomes for all networks can be obtained without data augmentation techniques.

**Table 2:** The global performance of the CNNs on the NINA and NIA datasets are reported. In addition, image improvement techniques are not active

IIQ not active					
Net	Data augmentation	ACC (min)	ACC (max)	ACC (mean)	ACC (sd)
*AlexNet*	None	0.68	**1**	***0.89***	0.05
	Yes	**0.76**	0.97	0.87	0.05
*DenseNet*	None	0.62	0.79	0.74	0.04
	Yes	0.41	0.88	0.73	0.08
*Google InceptionV3*	None	0.56	0.94	0.74	0.07
	Yes	0.32	0.71	0.55	0.07
*GoogleNet*	None	0.65	0.94	0.80	0.06
	Yes	0.30	0.76	0.55	0.09
*MobileNet*	None	0.50	0.91	0.75	0.09
	Yes	0.35	0.76	0.56	0.08
*ShuffleNet*	None	0.44	0.88	0.69	0.08
	Yes	0.26	0.74	0.52	0.10
*SqueezeNet*	None	0.38	1	0.55	0.11
	Yes	0.15	0.79	0.58	0.10
*VGG*	None	0.59	0.82	0.75	0.04
	Yes	0.59	0.82	0.73	0.05

**Fig. 6:**
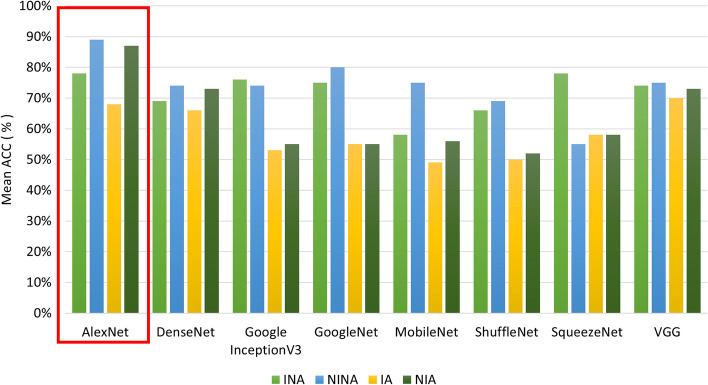
The global performance of the CNN on the four datasets

**Fig. 7:**
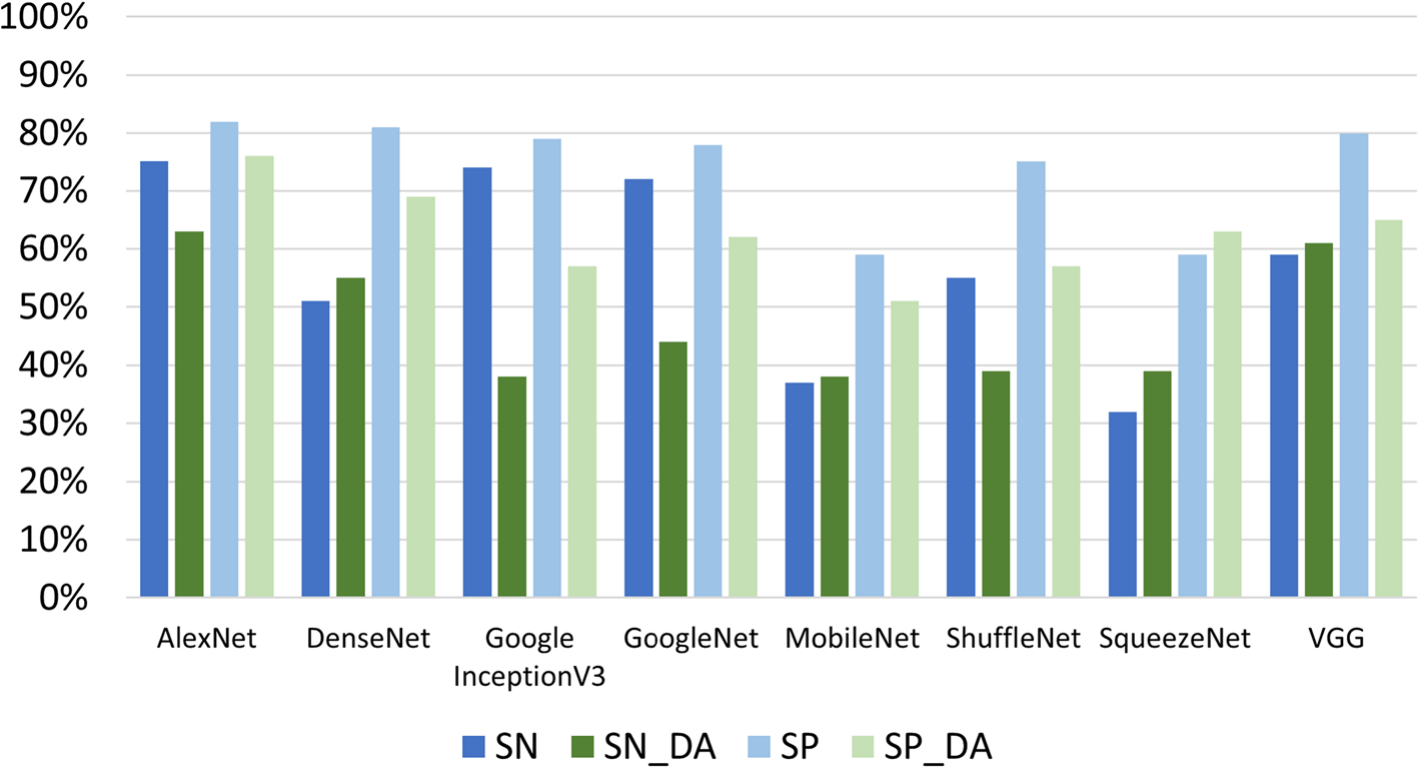
Comparison of SN and SP for the INA e IA datasets

**Fig. 8:**
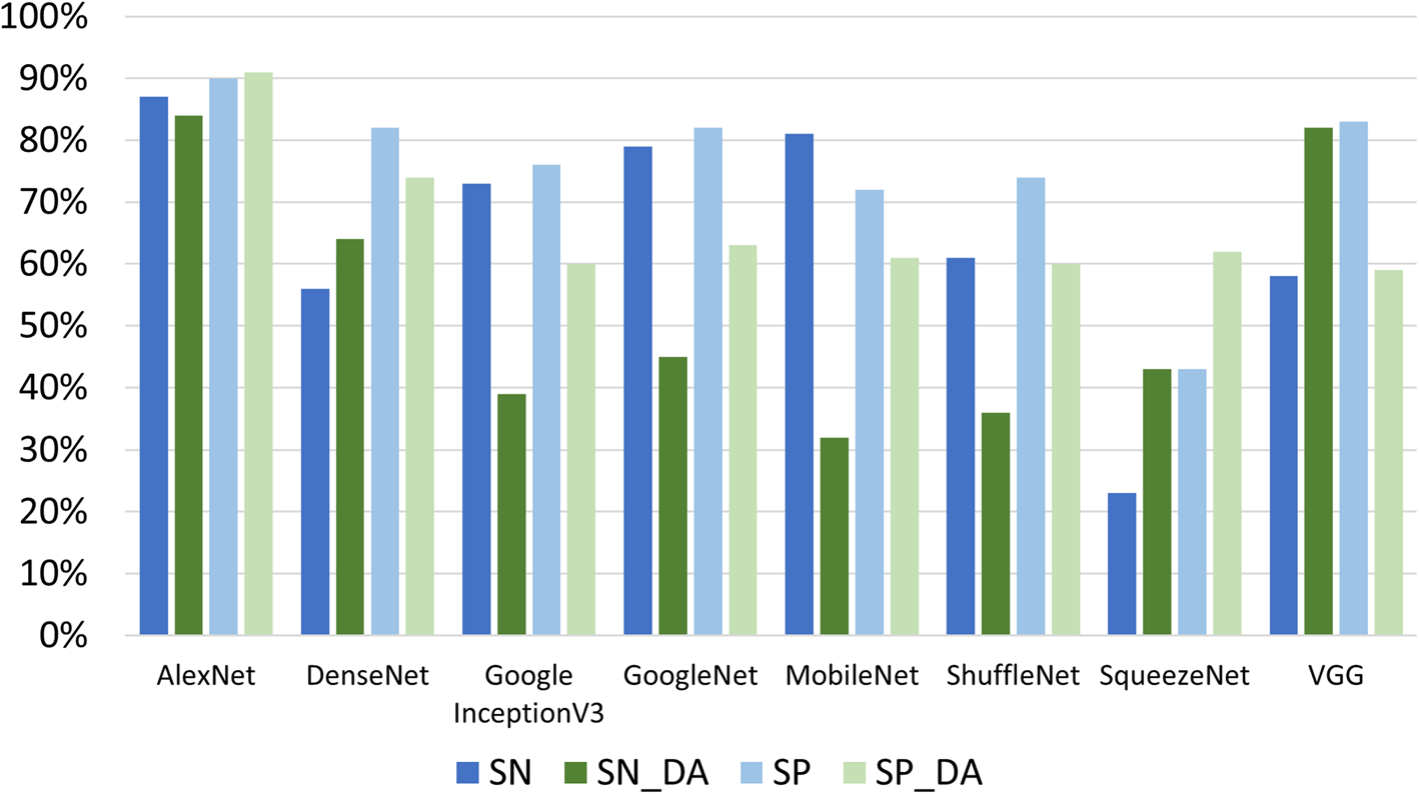
Comparison of SN and SP for the NINA e NIA datasets

In Fig. 6, a summarization of the results in terms of global performance is reported. The red box highlights that AlexNet is the network with the best performance in the four considered conditions (IA, INA, NIA, NINA). Table 3 (refering to the “IA” and “INA” datasets) and Table 4 (refering to the “NINA” and “NIA” datasets) report the standard metrics for evaluating the tested networks. Figures 7 and 8 report the table data graphically, where _[DA] represents data augmentation. As expected, the best results for SP and SN are obtained without data augmentation due to the previous accuracy results presented: we can see that experiments without data augmentation outperform all methods except SqueezeNet, which is the only exception; in SqueezeNet, SP and SN values increase with data augmentation.

However, in the context of MCIBCP, the FNR takes on more weight because it is directly related to the type 2 error. Therefore, the results reported in Table 3 and Table 4 suggest:

- SqueezeNet ensures the lowest FNR (0.13) on the “INA” dataset;
- AlexNet ensures the lowest FNR when used on the “NINA” dataset (0.13);
- DenseNet ensures the lowest FNR on the “IA” dataset (0.27);
- VGG ensures the lowest FNR on the “NIA” dataset (0.07).

Interestingly, even though SqueezeNet is confirmed as the worst network in global terms, it ensured the lowest FNR in at least one case. Therefore, SqueezeNet in the INA situation can be chosen to minimize type 2 errors.

**Table 3:** The FNR and the other metrics of the CNNs on the IA and INA datasets are reported. The image improvement techniques are active

IIQ active							
Net	Data augmentation	SN	SP	PPV	FDR	FNR	FPR
*AlexNet*	None	0.75	0.82	0.73	0.27	0.25	0.18
	Yes	0.63	0.76	0.65	0.35	0.37	0.24
*DenseNet*	None	0.51	0.81	0.78	0.22	0.34	0.19
	Yes	0.55	0.69	0.62	0.38	0.27	0.24
*Google InceptionV3*	None	0.74	0.79	0.68	0.32	0.26	0.21
	Yes	0.38	0.57	0.38	0.62	0.47	0.41
*GoogleNet*	None	0.72	0.78	0.67	0.33	0.28	0.22
	Yes	0.44	0.62	0.40	0.60	0.51	0.37
*MobileNet*	None	0.37	0.59	0.09	0.91	0.48	0.41
	Yes	0.38	0.51	0.59	0.41	0.53	0.32
*ShuffleNet*	None	0.55	0.75	0.67	0.33	0.41	0.25
	Yes	0.39	0.57	0.51	0.49	0.55	0.42
*SqueezeNet*	None	0.32	0.59	0.79	0.21	***0.13***	0.11
	Yes	0.39	0.63	0.42	0.58	0.42	0.36
*VGG*	None	0.59	0.80	0.71	0.30	0.26	0.20
	Yes	0.61	0.65	0.64	0.36	0.30	0.18

**Table 4:** The FNR and the other metrics of the CNNs on the NINA and NIA datasets are reported. The image improvement techniques are active

IIQ not active							
Net	Data augmentation	SN	SP	PPV	FDR	FNR	FPR
*AlexNet*	None	0.87	0.90	0.86	0.15	0.13	0.10
	Yes	0.84	0.91	0.87	0.14	0.16	0.09
*DenseNet*	None	0.56	0.82	0.77	0.23	0.29	0.18
	Yes	0.64	0.74	0.56	0.44	0.19	0.26
*Google InceptionV3*	None	0.73	0.76	0.62	0.38	0.27	0.24
	Yes	0.39	0.60	0.29	0.71	0.57	0.40
*GoogleNet*	None	0.79	0.82	0.72	0.28	0.21	0.18
	Yes	0.45	0.63	0.48	0.52	0.54	0.37
*MobileNet*	None	0.81	0.72	0.45	0.55	0.14	0.28
	Yes	0.32	0.61	0.29	0.71	0.37	0.38
*ShuffleNet*	None	0.61	0.74	0.60	0.40	0.35	0.26
	Yes	0.36	0.60	0.45	0.55	0.52	0.39
*SqueezeNet*	None	0.23	0.43	0.43	0.57	0.22	0.27
	Yes	0.43	0.62	0.41	0.59	0.41	0.37
*VGG*	None	0.58	0.83	0.76	0.24	0.27	0.17
	Yes	0.82	0.59	0.40	0.60	***0.07***	0.24

## Conclusion

Melanoma is a severe type of skin cancer responsible for about 99,780 new malignant diagnoses.^3^ However, if there is an early diagnosis, melanoma can be cured in most cases. Melanomas exist in many different shapes, sizes, and colours and affect people with all skin types. These characteristics are used by dermatologists to apply the ABCDE rules that can be used to estimate the degree of threat regarding a naevus. Unfortunately, nowadays, the last word regarding the malignancy of a lesion is delegated to the biopsy, which performs the histological analysis of the suspected lesion. Unfortunately, this state-of-the-art protocol can lead to delays in diagnosis and unnecessary invasive surgery in the case of false positives. In the case of non-detection of a melanoma, this false negative outcome could result in potentially fatal circumstances. In recent years, multiple computer-aided diagnosis (CAD) systems working on melanoma images have been proposed to speed up diagnosis. In addition, some results in the literature suggest that artificial intelligence techniques can outperform dermatologists in melanoma diagnosis, particularly CNN. These networks have been proven to give the most accurate and exact results for choosing between benign and malignant outcomes. If the accuracy of these CNN continues to grow in the future, unnecessary biopsies (type 1 error - false positive) will be avoided more and more, while needed biopsies (type 2 error - false negative) will be missed only a few times. In this complex context, where early melanoma treatment, minimizing false negative rates and providing easy-to-use tools to physicians is critical, our work aims to investigate the current CNN architectures available. In particular, we aimed to identify the CNN network structure that ensures the lowest FNR when used with Clinical Melanoma Images: nine CNNs, including Alexnet, DenseNet, GoogleNet Inception V3, GoogleNet, MobileNet, ShuffleNet, SqueezeNet, and VGG16 were evaluated. We started from the MED-NODE dataset, which includes 170 clinical photos (70 images of melanoma and 100 images of naevi) extracted from the digital image archive of the Department of Dermatology of the University Medical Center of Groningen (UMCG). Due to the small size of the dataset, we used image improvement and data augmentation techniques; four datasets (NINA, NIA, INA, IA) were generated to investigate the impact of data augmentation and image pre-processing on the final classification performance. The training, validation, and test sessions were executed on each dataset. Overall, all tested neural networks, with one exception, perform better without data augmentation, with a maximum accuracy of 0.78% achieved by AlexNet and SqueezeNet. In the absence of pre-processing and data augmentation, AlexNet performed best with 0.89%, 0.75% and 0.82% of accuracy, sensitivity, and specificity, respectively. In the context of MCIBCP, however, the FNR is more important than global accuracy because it is directly related to type 2 errors, which can result in life-threatening situations.The results suggest that the VGG CNN can ensure the lowest FNR at the expense of global accuracy, while AlexNet can ensure comparable FNR like VGG but with the highest global accuracy. Therefore, VGG and AlexNet were the CNNs that might be used to build a CAD system, FNR-driven and easy to use due to the capability to use clinical images instead of dermoscopic images. The remarkable results obtained with clinical images alone, whose quality is unquestionably lower than that of dermoscopic images, enable help in prevention in a situation where it is crucial. In the particular case of these experiments, the results highlight how the best values are often achieved without pre-processing techniques and data augmentation. The results strongly suggest the importance of using datasets that reflect a real scenario, avoiding adding artefacts and losing information from the images (such as skin around the lesion). In particular, the results strongly suggest the need for better segmentation masks that must correctly catch the skin part that can provide important information to the prediction model.

In summary, our findings highlight the significance of CAD systems in speeding up the diagnosis of melanoma, by focusing on clinical melanoma images instead of dermoscopic images, making the proposed CAD system more accessible and user-friendly for physicians. Also, we address the critical aspect of reducing FNR to prevent fatal outcomes. Among the weaknesses, we can consider the use of a small dataset size that may restrict the generalizability of the results. There is also a minimal examination or comparison of dermatologist performance, which would shed light on the advantages and disadvantages of the suggested CAD system. The reliability and applicability of the research in the area of melanoma detection would be improved by addressing these issues.

Finally, our results support what has already been discovered, which is that networks perform better when using the original images without any pre-processing [19, 42]. Additional research on this aspect might aid in understanding the motivation behind this behavior. Furthermore, future research could investigate local and global features relevant to melanoma, other neural networks, and different image pre-processing techniques in order to minimise the FNR while simultaneously maximising the global accuracy and the other metrics. Moreover, we plan to investigate dermoscopic contexts, referring to challenges such as ISIC (International Skin Imaging Collaboration). By exploring dermoscopic contexts and participating in challenges like ISIC, we seek to leverage the power of machine learning and computer vision techniques to enhance the capabilities of dermatologists in diagnosing skin conditions.

## Data Availability

The dataset analysed during the current study are available from https://www.cs.rug.nl/~imaging/databases/melanoma_naevi/

## Abbreviations

ACC: Accuracy
AI: Artificial Intelligence
AI: Artificial intelligence
AK: Actinic keratosis
CAD: Computer-aided diagnosis
BCC: Basal cell carcinoma
BD: Bowen diseas
CNN: Convolutional neural network
CT: Computed tomography
DA: Data augmentation
DF: Dermatofribroma
DL: Deep learning
FDR: False discovery rate
FNR: False negative rate
FPR: False positive rate
FRCNN: Faster region-based CNN
GPU: Graphics processing unit
IoMD: Internet of medical devices
IoMT: Internet of medical things
IoT: Internet of Things
MCIBCP: Melanoma clinical image binary classification problem
ML: Machine learning
MM: Malignant melanoma
MN: Melanocytic naevus
MRI: Magnetic resonance imaging
NCN: Naevus cell naevus
NN: Neural network
PET: Positron emission tomography
PVV: Positive predicted value
SCC: Squamous cell carcinoma
SK: Seborrhoeic keratosis
SP: Specificity
TL: Transfer learning
UMCG: University Medical Center Groningen

## Footnotes

1 https://www.cancer.org/cancer/melanoma-skin-cancer/about/key-statistics.html.

2 https://www.mathworks.com/products/matlab.html.

3 https://www.cancer.org/cancer/melanoma-skin-cancer/about/key-statistics.html.
